# Efficacy and tolerability of lamivudine plus dolutegravir compared with lamivudine plus boosted PIs in HIV-1 positive individuals with virologic suppression: a retrospective study from the clinical practice

**DOI:** 10.1186/s12879-018-3666-8

**Published:** 2019-01-17

**Authors:** Alberto Borghetti, Francesca Lombardi, Roberta Gagliardini, Gianmaria Baldin, Arturo Ciccullo, Davide Moschese, Arianna Emiliozzi, Simone Belmonti, Silvia Lamonica, Francesca Montagnani, Elena Visconti, Andrea De Luca, Simona Di Giambenedetto

**Affiliations:** 1 Institute of Clinical Infectious Diseases, Catholic University of Sacred Heart, Policlinico Gemelli, Rome, Italy; 2 Infectious Diseases Unit, Siena University Hospital, Viale Mario Bracci, 53100 Siena, Italy

**Keywords:** HIV, Antiretroviral therapy, Maintenance therapy, Dual therapy, Lamivudine, Dolutegravir, Atazanavir/ritonavir, Darunavir/ritonavir

## Abstract

**Background:**

Direct comparisons between lamivudine plus bPIs and lamivudine plus dolutegravir as maintenance strategies in virologically-suppressed HIV positive patients are lacking.

**Methods:**

Time to treatment discontinuation (TD) and virological failure (VF) were compared in a cohort of HIV+ patients on a virologically-effective ART starting lamivudine with either darunavir/r, atazanavir/r or dolutegravir. Changes in laboratory parameters were also evaluated.

**Results:**

Four-hundred-ninety-four patients were analyzed (170 switching to darunavir/r, 141 to atazanavir/r, 183 to dolutegravir): median age was 49 years, with 8 years since ART start. Groups differed for age, HIV-risk factor, time since HIV-diagnosis and on ART, previous therapy and reasons for switching.

Estimated proportions free from TD at week 48 and 96 were 79.8 and 48.3% of patients with darunavir/r, 87.0 and 70.9% with atazanavir/r, and 88.2 and 82.6% with dolutegravir, respectively (*p* < 0.001). Calendar years, HIV-risk factor, higher baseline cholesterol and an InSTI-based previous regimen predicted TD, whereas lamivudine+dolutegravir therapy and previous tenofovir use were protective. VF was the cause of TD in 6/123 cases with darunavir/r, 4/97 with atazanavir/r and 3/21 with dolutegravir. Other main reasons for TD were: toxicity (43.1% with darunavir/r, 39.2% with atazanavir/r, 52.4% with dolutegravir), further simplification (36.6% with darunavir/r, 30.9% with atazanavir/r, 14.3% with dolutegravir). Incidence of VF did not differ among study groups (*p* = 0.747). No factor could predict VF.

Lipid profile improved in the dolutegravir group, whereas renal function improved in the bPIs groups.

**Conclusions:**

In real practice, a switch to lamivudine+dolutegravir showed similar efficacy but longer durability than a switch to lamivudine+bPIs.

## Background

Combination antiretroviral therapy (ART) has dramatically decreased the incidence of AIDS-defining events in the HIV-infected population [1]. International guidelines [2, 3] recommend a prompt initiation of ART independently from CD4 cell count in order to reduce morbidity and mortality associated with HIV infection. Conversely, even if non-AIDS defining events are reduced by the immediate initiation of ART [4] the beneficial effects of long-term therapy on the burden of some non-AIDS related conditions (such as cardiovascular disease) remain to be established. Mathematical models [5] forecast that, in the next few decades, age-related comorbidities will become increasingly relevant in the management of HIV-infected individuals, especially due to an increased burden of cardiovascular diseases, diabetes and chronic kidney disease. Moreover, the indication to treat all HIV-infected patients regardless of their CD4 counts [4] raises the issue of the long-term treatment-related toxicities, such as those related to nucleoside reverse transcriptase inhibitors, NRTIs [6], and patients’ adherence [7]. Finally, the increasing prevalence of HIV infection, the improved life expectancy of the infected population, and the increased projected lifetime healthcare costs also prompt the need for new treatment paradigms [8].

After achieving a stable virological suppression, treatment simplification with two-drugs regimens is a switch strategy considered by both US [2] and European [3] guidelines in order to manage or prevent ART toxicity or in case of comorbidities, drug-drug interactions, or when treatment simplification is required. Among these strategies, lamivudine plus boosted protease inhibitors (bPIs) are supported by the most robust evidence [9–13]. However, due to the impact of bPIs on the metabolic profile [9–13] and the potential for drug-drug interactions of boosting agents [14], new treatment options are under investigation. Recent observational studies [15–17] and a pilot study [18] suggest that switching to lamivudine plus dolutegravir could be a feasible option, overcoming some disadvantages of bPIs. The virological efficacy of this combination, that also emerged in naïve patients [19], and the protective effect of the M184I/V mutation on the emergence of resistance mutations against dolutegravir [20], makes it a potential option in highly treatment-experienced patients with a long history of HIV disease and previous virological failures to NRTIs but not to integrase strand transfer inhibitors (InSTIs).

So far, direct comparisons between lamivudine plus bPIs and lamivudine plus dolutegravir as maintenance strategies in virologically-suppressed HIV positive patients are lacking. Therefore, we aimed to compare the virological efficacy, safety and durability of lamivudine plus either atazanavir/ritonavir (r), darunavir/r or dolutegravir in clinical practice.

## Methods

Three groups of ART-treated HIV-positive adult (≥ 18 years) patients, followed-up in a single clinical center, starting a two-drug regimen with lamivudine plus either atazanavir/r, darunavir/r or dolutegravir in a period ranging from 2008 to 2017 were retrospectively evaluated. Switching to a dual regimen was driven by current toxicity issues or need for prevention of future toxicities, and all patients had undetectable viral load at the time of regimen initiation (HIV-RNA < 50 copies/mL). Due to increasing knowledge about treatment toxicities and a greater availability of different treatment choices over time, reasons for beginning a lamivudine-based dual regimen changed in different study periods. In order to observe these changes, no exclusion criteria were selected about study population, with the exception of hepatitis B surface antigen-positive serostatus. The choice of this criterion was mainly driven by the possibility of finding patients who could inadvertently have had their three drug-regimens switched to lamivudine-based dual therapy without checking HBsAg serostatus and that could subsequently have undergone treatment discontinuation at the time of serological test availability.

Data analyzed in the present study were selected from an internal observational database, which collects main clinical and demographic characteristics of every patient who gave informed consent to personal data record since the time of HIV diagnosis. The creation of the database was approved by the “Fondazione Policlinico Gemelli” Ethics Committee (protocol number: 10978/15).

Main outcomes measures were time free from treatment discontinuation (TD, i.e. interruption of one or both study drugs or intensification of study regimen) and time free from virological failure (VF, as defined by 2 consecutive HIV-RNA ≥50 copies/mL or a single HIV-RNA ≥1000 copies/mL), estimated by the Kaplan-Meier method and compared between groups by the log-rank test. The follow up was censored at TD, death, loss to follow-up, or last available clinical data (until April 30, 2017), whichever occurred first. For the VF analysis, TD was used as an additional censoring criterion. Predictors of TD and VF were evaluated by Cox regression.

Secondary endpoints were the evaluation and comparison of changes in CD4 count, blood lipids and estimated glomerular filtration rate (eGFR, according to MDRD study equation) at different times (baseline, week 24 and 48) by using generalized linear models for repeated measures. Predictors of changes were identified by linear regression analysis.

## Results

Four-hundred-ninety-four patients were eligible for the analysis: 170 started lamivudine plus darunavir/r, 141 lamivudine plus atazanavir/r and 183 lamivudine plus dolutegravir. Overall, 348 (70.4%) were male, with 49 years of median age, 12 years since HIV diagnosis and 8 years on ART. Characteristics of the study population are summarized in Table 1.

**Table 1:** Characteristics of study population at baseline (*N* = 484)

Variables	Darunavir/r *N* = 170	Atazanavir/r *N* = 141	Dolutegravir *N* = 183	*p*
Age*	48 (40–54)	48 (43–53)	51 (43–57)	0.008
Male sex	126 (74.1)	95 (67.4)	127 (69.4)	0.399
Caucasians	157 (92.4)	136 (96.5)	164 (89.6)	0.165
HIV risk factor				0.043
Heterosexual	74 (43.5)	68 (48.2)	83 (45.4)	
MSM	84 (49.4)	49 (34.8)	80 (43.7)	
IDUs	11 (6.5)	24 (17.0)	19 (10.4)	
Other	1 (0.6)	0	1 (0.5)	
CDC stage C	48 (28.2)	34 (24.1)	53 (29.0)	0.592
HCV positive serostatus	18 (10.6)	25 (17.7)	26 (14.2)	0.193
Nadir CD4 count (cells/μL)*	219 (64–311)	194 (62–293)	194 (64–284)	0.741
Zenith HIV-RNA (log_10_ copies/mL)*	4.90 (4.36–5.40)	5.02 (4.48–5.39)	4.93 (4.34–5.40)	0.687
CD4 count at baseline (cells/μL)*	617 (484–793)	616 (509–786)	630 (500–800)	0.671
Years since HIV diagnosis*	9 (5–17)	11 (4–18)	14 (8–20)	< 0.001
Years on antiretroviral therapy*	8 (3–14)	7 (3–13)	12 (5–18)	< 0.001
Previous virological failure	91 (53.5)	78 (55.3)	94 (51.4)	0.775
Previous M184 V resistance mutation	22 (12.9)	17 (12.1)	16 (8.7)	0.419
Years of viral suppression at baseline*	5 (2–8)	4 (2–7)	8 (4–11)	< 0.001
Previous regimen:				< 0.001
2NRTI + bPI	80 (47.1)	125 (88.7)	14 (7.7)	
2NRTI + NNRTI	20 (11.8)	4 (2.8)	34 (18.6)	
2NRTI + INI	24 (14.1)	1 (0.7)	32 (17.5)	
Two-drug regimen	34 (20.0)	8 (5.7)	99 (54.1)	
- Lamivudine plus bPI	32 (18.8)	8 (5.7)	89 (48.6)	
- bPI plus NNRTI, InSTI or MVC	2 (1.2)	0 (0)	6 (3.3)	
- Other regimen	0 (0)	0 (0)	4 (2.2)	
Other	12 (7.1)	3 (2.1)	4 (2.2)	
TDF in previous regimen	97 (57.1)	119 (84.4)	65 (35.5)	< 0.001
Reasons for switch to dual regimen:				< 0.001
Simplification	86 (50.6)	100 (70.9)	57 (31.1)	
Dyslipidemia	8 (4.7)	7 (5.0)	60 (32.8)	
GI toxicity	4 (2.4)	2 (1.4)	13 (7.1)	
Liver toxicity	11 (6.5)	0	4 (2.2)	
Renal toxicity	28 (16.5)	12 (8.5)	15 (8.2)	
Bone toxicity	3 (1.8)	3 (2.1)	12 (6.6)	
CNS toxicity	1 (0.6)	0	3 (1.6)	
Other toxicity	14 (8.2)	4 (2.8)	7 (3.8)	
DDI	2 (1.2)	0	6 (3.3)	
Other	13 (7.6)	13 (9.2)	6 (3.3)	
Years on previous regimen*	2 (1–4)	3 (1–5)	2 (1–3)	0.530
Study period:				< 0.001
2008–2013	37 (21.8)	88 (62.4)	0	
2013–2014	91 (53.5)	33 (23.4)	0	
2014–2015	34 (20.0)	12 (8.5)	77 (42.1)	
2015–2017	8 (4.7)	8 (5.7)	106 (57.9)	

Note: *bPI* boosted protease inhibitor, *TDF* tenofovir, *MVC* maraviroc, *GI* gastrointestinal, *CNS* central nervous system, *DDI* drug-drug interaction

*Continuous variables (interquantile range)

**Fig. 1:**
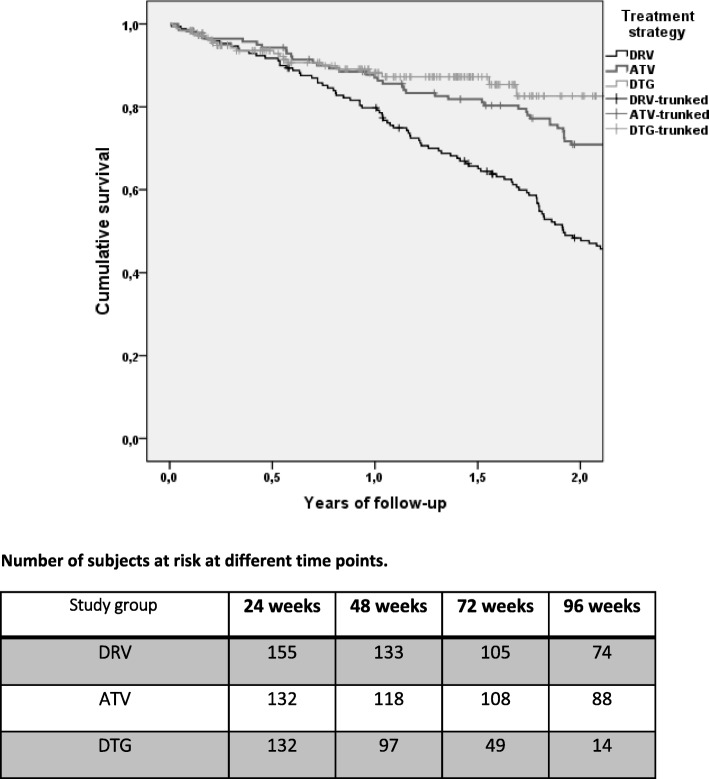
Estimated proportions surviving without TD divided by regimen type (log-rank *p* < 0.001)

Over 333.2 patient-years follow-up (PYFU) there were 123 discontinuations in the darunavir/r group (incidence rate, IR, 36.9 per 100 PYFU), 97 over 461.1 PYFU (21.0 per 100 PFU) in the atazanavir/r group and 21 over 189.1 PYFU (11.1 per 100 PYFU) in the dolutegravir group. Estimated probabilities of remaining free from TD at week 48 and 96 were, respectively, 79.8% (95% confidence interval, CI, 73.7–85.9%) and 48.3% (95% CI 40.7–55.9%) with darunavir/r, 87.0% (95% CI 81.3–92.7%) and 70.9% (95% CI, 63.3–78.5%) with atazanavir/r, 88.2% (95% CI 82.9–93.5%) and 82.6% (95% CI 74.4–90.8%) with dolutegravir (log-rank *p* < 0.001). Between-group comparisons showed a significant difference between darunavir/r and atazanavir/r (*p* < 0.001) and between darunavir/r and dolutegravir (*p* < 0.001), but no differences between atazanavir/r and dolutegravir (*p* = 0.252; see Fig. 1). Considering that a not negligible proportion of the patients switching to lamivudine and darunavir and, above all, to lamivudine and dolutegravir were already on a dual regimen (mostly lamivudine-based), a secondary analysis excluding these patients on a previous dual therapy was performed. Also in this case, the probability of remaining on lamivudine plus dolutegravir at 48 weeks (89.0, 95% CI 81.5–96.5%) and 96 weeks (76.3, 95% CI 52.4–100.0%) was higher compared to the probability of remaining with lamivudine and darunavir/r (82.1, 95% CI 75.6–88.6% at week 48; 40.6, 95% CI 40.6–57.8% at week 96; log-rank *p* = 0.040), but not with lamivudine and atazanavir/r (88.6, 95% CI 83.1–94.1% at week 48; 72.5, 95% CI 64.7–80.3% at week 96; log-rank *p* = 0.745).

After adjusting for baseline factors that were significantly different among groups, calendar period of regimen initiation (2015 or later versus 2013 and before), HIV-risk factor (injecting drug use versus heterosexual contacts), higher baseline cholesterol and the use of a InSTI-based three-drug regimen before switch (versus a bPI-based three-drug regimen) were associated with TD, whereas dolutegravir use (versus bPIs), switch from tenofovir (versus other backbones) and duration of the previous regimen were protective factors (see Table 2). When considering the two-drug regimens separately in the same multivariate model, dolutegravir was independently associated with a lower hazard of discontinuation when compared with both darunavir/r (aHR: 0.15, 95% CI 0.08–0.27; *p* < 0.001) and atazanavir/r (aHR: 0.19, 95% CI 0.10–0.39; *p* < 0.001).

**Table 2:** Predictors of TD. Univariable and multivariable Cox regression

Variables	HR (95% CI)	*p*	aHR (95% CI)	*p*
Age (per 10 years more)	1.03 (0.91–1.15)	0.675	1.04 (0.91–1.19)	0.565
HIV risk factor:				
Heteosexual	Ref		Ref	
MSM	1.14 (0.87–1.50)	0.358	1.25 (0.93–1.68)	0.143
IDUs	1.39 (0.93–2.08)	0.112	1.75 (1.13–2.70)	0.011
Other	1.72 (0.24–12.38)	0.590	0.84 (0.11–6.73)	0.870
Previous regimen:				
2NRTI + bPI	Ref		Ref	
2NRTI + NNRTI	1.09 (0.65–1.81)	0.754	1.13 (0.66–1.93)	0.662
2NRTI + InSTI	1.87 (1.21–2.90)	0.005	2.16 (1.30–3.60)	0.003
Two-drug regimen	1.15 (0.79–1.67)	0.466	0.64 (0.37–1.10)	0.103
Other	1.43 (0.79–2.59)	0.233	0.84 (0.41–1.71)	0.631
Switch from TDF-containing regimen	0.72 (0.54–0.95)	0.019	0.59 (0.39–0.88)	0.010
Reasons for switch to dual regimen:				
Simplification	Ref		Ref	
Dyslipidemia	0.71 (0.41–1.21)	0.207	0.74 (0.40–1.36)	0.332
GI toxicity	1.04 (0.42–2.56)	0.929	1.12 (0.43–2.87)	0.820
Liver toxicity	2.18 (1.14–4.16)	0.019	1.50 (0.71–3.17)	0.286
Renal toxicity	1.17 (0.76–1.78)	0.475	1.14 (0.73–1.80)	0.565
Bone toxicity	1.45 (0.60–3.62)	0.405	1.12 (0.3–2.91)	0.813
CNS toxicity	1.21 (0.17–8.71)	0.849	1.94 (0.25–14.87)	0.524
Other toxicity	2.98 (1.83–4.84)	< 0.001	2.16 (1.27–3.70)	0.005
DDI	2.24 (0.71–7.09)	0.169	1.82 (0.54–6.12)	0.330
Other	1.37 (0.84–2.25)	0.207	1.25 (0.74–2.13)	0.408
Time on previous regimen (per 1 year more)	0.98 (0.92–1.04)	0.547	0.92 (0.85–0.99)	0.021
eGFR at baseline (per 10 mL/min/1.73 m more)	1.06 (1.00–1.11)	0.039	1.05 (0.99–1.11)	0.102
Total cholesterol at baseline (per 10 mg/dL more)	1.03 (1.00–1.06)	0.058	1.04 (1.01–1.07)	0.012
Years on antiretroviral therapy	1.01 (0.99–1.03)	0.208	1.01 (0.99–1.04)	0.330
Study period:				
2008–2013	Ref		Ref	
2013–2014	2.04 (1.44–2.87)	< 0.001	1.98 (1.37–2.88)	< 0.001
2014–2015	2.25 (1.47–3.43)	< 0.001	4.88 (3.00–7.94)	< 0.001
2015–2017	2.18 (1.20–3.97)	< 0.001	8.45 (4.12–17.31)	< 0.001
Study treatment with Dolutegravir (versus bPIs)	0.50 (0.31–0.80)	0.004	0.15 (0.08–0.28)	< 0.001

Note: *bPI* boosted protease inhibitor, *TDF* tenofovir, *GI* gastrointestinal, *CNS* central nervous system, *DDI* drug-drug interaction, *eGFR* estimated glomerular filtration rate

Reasons for TD varied among groups (*p* < 0.001). With darunavir/r, 6 (4.9%) interruptions were due to VF, 53 (43.1%) to toxicity (35 cases of dyslipidemia, 11 cases of gastro-intestinal toxicity, 1 case of renal toxicity and 1 of neurological toxicity, 5 of unspecified patients intolerance), 45 (36.6%) due to further simplification, 7 (5.7%) due to drug interactions and 12 (9.8%) due to other/unknown reasons. With atazanavir/r discontinuations occurred for VF in 4 (4.1%) cases, toxicity in 38 (39.2%) (10 dyslipidemia, 1 gastro-intestinal toxicity, 15 liver toxicity, 9 renal toxicity, 3 other or unspecified toxicity), simplification in 30 (30.9%), drug interactions in 8 (8.2%), other/unknown reasons in 17 (17.5%). With dolutegravir, reasons for TD were VF in 3 (14.3%) cases, toxicity in 11 (52.4%) (3 gastro-intestinal toxicity, 1 liver toxicity, 6 neuropsychological toxicity, 1 unspecified intolerance), simplification in 3 (14.3%) and unspecified reasons in 4 (19.0%).

VF occurred in 11 patients over 319.2 PYFU (IR 3.5 per 100 PYFU) with darunavir/r, with estimated probabilities of remaining free from VF of 95.4% (95% CI 92.1–98.7) and 92.5% (95% CI 88.0–97.0) at week 48 and 96, respectively. Ten VF over 441 PYFU (IR 2.3 per 100 PYFU) occurred with atazanavir/r, with estimated probabilities of remaining free from VF of 96.1% (95% CI 92.8–99.4) and 95.1% (95% CI 91.2–99.0) at week 48 and 96, respectively. With dolutegravir, 5 VF occurred over 188 PYFU (IR 2.7 per 100 PYFU): the estimated probabilities of remaining free from VF were 97.5% (95% CI 94.8–100) and 94.5% (95% CI 89.6–99.4) at week 48 and 96, respectively. No difference in the time to VF was detected among study groups (log-rank *p* = 0.747). Potential exposure variables associated to VF were analyzed: nor immunological parameters (baseline and nadir CD4+ count), nor viral factors (especially zenith HIV-RNA or the presence of M184 V/I at historical genotypes), nor HCV coinfection, time since HIV diagnosis and years of exposure to antiretrovirals, could predict an increased risk of VF.

Of 494 patients, 341 had week 24 and 48 follow-up CD4 counts, 295 total cholesterol (TC) and HDL, 362 triglycerides, 369 eGFR. At baseline, groups differed for eGFR levels only, these were slightly higher in the dolutegravir group (*p* < 0.001).

Absolute CD4 counts significantly increased in all groups from baseline to 48 weeks (mean + 54 cells/μL; within-groups effect test-p < 0.001), with no significant changes in CD4/CD8 ratio. No differences among groups were seen at different time points (between-groups effect test-*p* = 0.530) and no time-group interaction was found (*p* = 0.477). A previous AIDS-event (versus none, mean difference in change − 3.40 cells/μL, 95% CI -5.09; − 1.71; *p* < 0.001) and baseline CD4 count (per 100 cells/μL higher, mean difference in change: − 98.41 cells/μL, 95% CI -98.72; − 98.11; *p* < 0.001) independently predicted CD4 change at week 48, after adjusting for type of dual regimen and nadir CD4 count.

A decrease in TC/HDL ratio emerged with lamivudine plus dolutegravir (− 0.39 at week 24, *p* < 0.001; − 0.33 at week 48, *p* = 0.001) compared with lamivudine plus bPIs (time-group interaction-effect test-*p* = 0.006): the differences in mean change in TC/HDL between dolutegravir and atazanavir/r (− 0.50; *p* = 0.047) and between dolutegravir and darunavir/r (− 0.53; *p* = 0.012) were significant only at week 24. The use of dolutegravir (versus bPIs, mean difference in change: -0.32, 95% CI -0.61; − 0.02; *p* = 0.036) and baseline TC/HDL values (per 1 unit higher, mean difference in change: -0.43, 95% CI -0.51; − 0.35; *p* < 0.001) predicted a decreased TC/HDL at week 48, after adjusting for use of cholesterol-lowering therapy after baseline, time to start of cholesterol-lowering therapy, previous tenofovir use and cumulative time on ART.

Despite a greater use of triglycerides-lowering therapies since or after baseline in the bPIs groups compared with dolutegravir group (*p* = 0.003), triglycerides only decreased in the latter (mean change from baseline − 32 mg/dL at week 24, p < 0.001; − 36 mg/dL at week 48, *p* < 0.001), with a significant time-group interaction (*p* < 0.001). The difference in mean change of triglycerides between dolutegravir and both bPIs emerged at both week 24 (*p* < 0.001) and week 48 (p = 0.003). Baseline triglycerides values (per 1 mg/dL higher, mean difference in change: − 0.43 mg/dL, 95% CI -0.50; − 0.36; *p* < 0.001) and the use of dolutegravir (versus bPIs, mean difference in change: − 25.13 mg/dL, 95% -42.43; − 7.82; *p* = 0.005) independently predicted a decrease in triglycerides at week 48, whereas previous tenofovir use (versus other backbones, mean difference in change: + 25.90 mg/dL, 95% CI + 9.42; + 40.38; *p* = 0.002) predicted their increase, independently from the start and time to start of a triglycerides-lowering therapy, history of myocardial infarction and cumulative time on ART.

Estimated GFR improved in the bPIs groups (mean change + 5 mL/min/1.73 m^2^, p = 0.005 at week 48 with darunavir/r; + 3 mL/min/1.73 m^2^ at week 48, *p* = 0.028 with atazanavir/r), with a significant time-group interaction (*p* < 0.001). The eGFR decreased in the first 24 weeks of dolutegravir use (mean change − 13 mL/min/1.73 m^2^, *p* < 0.001) and stabilized thereafter. Age (per 10 years older, mean difference in change: − 4.34 mL/min/1.73 m^2^, 95% CI -5.93, − 2.76; p < 0.001), baseline eGFR (per 1 mL/min/1.73 m^2^ higher, mean difference in change: − 0.39 mL/min/1.73 m^2^, 95% CI -0.45; − 0.33; *p* < 0.001) and the use of dolutegravir (versus bPIs, mean difference in change: − 12.88 mL/min/1.73 m^2^, 95% CI -16.97; − 8.79; *p* < 0.001) were negatively associated with the change in eGFR at 48 weeks, after adjusting for anti-HCV positive serostatus, previous use of tenofovir, cumulative time on ART and PIs and duration of virologic suppression at baseline.

## Discussion

A recent meta-analysis on lamivudine plus bPIs in patients with stable viral suppression confirmed the non-inferior virological efficacy of these strategies compared with standard three-drug regimens [21]. However, due to the increasing prevalence of comorbid conditions (especially cardiovascular and metabolic disorders) of people aging with HIV and to issues related to drug interactions, dual therapies with bPIs have lost part of their role as maintenance therapies in favor of more tolerable dual regimens. Particularly, two randomized trials about the use of rilpivirine and dolutegravir in patients who achieved viral control found no differences in the virological efficacy of this strategy when compared to standard therapy [22] and strongly support its role as a maintenance therapy. However, dietary constriction and some important drug interactions could represent a limit of this strategy, that would not be present by using the combination of lamivudine plus dolutegravir. In a randomized pilot clinical trial [23], this strategy was also non-inferior to continuation of standard three-drug maintenance therapy. Due to its favorable metabolic profile, the lack of major toxicities and the low projected costs, if ongoing trials will confirm its efficacy, the combination of lamivudine plus dolutegravir could change the management of HIV-infection in next years, as two systematic review on this subject also evidenced [24, 25].

Besides randomized trials, assessing the feasibility of lamivudine plus dolutegravir in clinical practice, especially in comparison with the other approved lamivudine-based simplification strategies, is of paramount importance. In our cohort lamivudine-based two-drug therapies in HIV-positive patients with suppressed HIV-RNA showed a good virological efficacy: the cumulative incidence of VF was less than 3.5 per 100 PYFU, without differences among study groups. This finding is in agreement with previous trials [10, 12, 13] and observational studies [15, 17, 18], including the preliminary data on lamivudine plus dolutegravir showing its high virological efficacy and good tolerability. In a multicenter cohort [15] of 110, virologically-suppressed patients on first line therapy, only one had a pre-defined VF; another cohort study, including 27 heavily treatment-experienced, virologically-suppressed patients [18], did not detect any VF after 96 weeks, despite the presence of M184 V mutation in 63% of the study population; finally, an Italian study [17] on 94 treatment-experienced patients on stable cART and virological suppression, switching to lamivudine plus dolutegravir, did not observe VF after 24 weeks of follow-up. In line with these reports, our results confirm the efficacy of lamivudine plus dolutegravir in different clinical contexts.

Here, for the first time, we present how the efficacy of different lamivudine-based dual therapies may impact in clinical practice. Findings from the dolutegravir group are reassuring but should be interpreted with caution, given that more than half of patients were already on a previous lamivudine-based dual regimen, suggesting that this was a selected sample of patients (however, the exclusion of patients switching from a previous dual regimen did not seem to affect the results of the main analysis). Moreover, follow-up time of patients on dolutegravir were shorter than follow-up of patients on lamivudine plus PIs: at week 96, only 14 patients with dolutegravir were still on study, compared with 74 on darunavir/r and 88 on atazanavir/r, indicating the reduced reliability of data after the first 48 weeks of treatment for lamivudine plus dolutegravir.

The main reason for TD was drug-related toxicity in all groups, but our data seem to underline an overall better tolerability of dolutegravir. Indeed, dolutegravir-treated group was independently associated with a lower discontinuation risk as compared with both darunavir/r- and atazanavir/r-treated groups. However, a series of possible study bias should be considered before drawing conclusions: some baseline characteristics also related to TD, namely the injecting drug use as a HIV-risk factor, the presence of dyslipidemia, and the previous regimen, were statistically different among groups. Patients switching from an InSTI-based regimen were at higher risk of TD. As previously found in a multicenter cohort study [11], this result could be ascribed to a worse tolerability and worse metabolic profile of bPIs as compared with InSTI. Of note, patients on lamivudine and darunavir/r mainly switched from a Insti-based three-drug regimen, and this could have emphasized the worsening of blood lipids commonly seen after switch to a PI-based regimen.

Conversely, tenofovir discontinuation was protective against TD, likely reflecting a loss of renal or bone toxicity, as observed in previous trials [10, 13], or a higher patients’ satisfaction with a less-drug regimen compared with standard three-drug regimens, that in our cohort mostly included tenofovir disoproxil fumarate. Almost 85% of patients on lamivudine plus atazanavir/r (compared with 57% of patients on lamivudine plus darunavir/r) were on a previous TDF-containing regimen and this could also have affected the lower rate of discontinuation of atazanavir/r compared to darunavir/r.

Interestingly, more recent study calendar periods were increasingly associated to the risk of TD, indicating a higher propensity to change, presumably related to the availability of more alternative regimens during more recent years. Considering the more recent introduction of dolutegravir and the shorter follow-up of patients on lamivudine and dolutegravir compared with patients on lamivudine plus PIs, prospective studies are needed to fully assess the durability of this strategy over time, especially in comparison with newer treatment strategies.

Blood lipids profile significantly improved after switching to lamivudine plus dolutegravir: the use of dolutegravir was associated with a reduction of TC/HDL ratio and triglycerides at week 48, independently from baseline values, use of lipid-lowering therapy and a previous tenofovir-containing regimen. Renal function apparently declined in the dolutegravir group at week 24 but stabilized thereafter, whereas an increase in eGFR was observed in the bPIs groups, independently from a previous tenofovir use, as already reported in the ATLAS-M trial [10]. These findings can be explained by the loss of interaction between bPIs and tenofovir disoproxil fumarate, that seems to accelerate the eGFR decline [26], more than the single effect of tenofovir withdrawal. It is also known that dolutegravir can lead to an initial non progressive increase in serum creatinine by inhibiting the renal organic cation transporter 2 (OCT2) [27], which could have further increased the difference in eGFR changes among study groups.

## Conclusions

To our knowledge this is the first observational study aiming to compare the durability of lamivudine-based two-drug regimens. Further strengths of our study are represented by the large sample size, the relatively long median follow-up time (1.6 years) and the lack of exclusion criteria that could have had an impact on efficacy of dual regimens (e.g., zenith HIV-RNA > 100.000 copies/mL, nadir CD4 count< 200 cells/μL). Of course, our study has important limitations, particularly in relation to its retrospective design and the lack of randomization, that hamper direct evaluation of differences among lamivudine-based dual regimens and that precluded to interpret the global efficacy of these simplification strategies. The different characteristics of study groups at baseline, partly explained by the different periods of enrolment, lead to the emergence of several important confounders: although we used multivariate models to address these issues, the strength and the generalization of our conclusions are limited.

Keeping that in mind, our study adds further proof that lamivudine plus dolutegravir could represent a potential optimization strategy in virologically-suppressed patients. The improvement of the blood lipids profile can be considered another advantage of this strategy: given the increased frequency of age-related comorbidities in the HIV-infected population, lamivudine plus dolutegravir could play an important role in the next years to reduce cardiovascular diseases in this setting. Longer follow-up studies are needed to verify this hypothesis.

## Abbreviations

ART: Combination antiretroviral therapy
bPIs: Boosted protease inhibitors
eGFR: estimated glomerular filtration rate
InSTIs: Integrase strand transfer inhibitors
NRTIs: Nucleoside reverse transcriptase inhibitors
OCT2: Organic cation transporter 2
TC: Total cholesterol
TD: Treatment discontinuation
VF: Virological failure

## References

[1] Broder S (2010). The development of antiretroviral therapy and its impact on the HIV-1/AIDS pandemic. Antivir Res.

[2] Panel on Antiretroviral Guidelines for Adults and Adolescents. Guidelines for the use of antiretroviral agents in HIV-1-infected adults and adolescents. Department of Health and Human Services. . (Updated 17 October 2017. Accessed on 22 Aug 2017). Available at http://www.aidsinfo.nih.gov/ContentFiles/AdultandAdolescentGL.pdf.

[3] EACS Guidelines version 8.2, January 2017. Available at http://www.eacsociety.org/files/guidelines_9.0-english.pdf.

[4] Lundgren JD, Babiker AG, Gordin F, Emery S, Grund B, Sharma S, Avihingsanon A, Cooper DA, Fatkenheuer G, Llibre JM (2015). Initiation of antiretroviral therapy in early asymptomatic HIV infection. N Engl J Med.

[5] Smit M, Cassidy R, Cozzi-Lepri A, et al. Quantifying the future clinical burden of an ageing HIV positive population in Italy. a mathematical modelling study. HIV Drug Therapy. 23–26 October 2016, Glasgow, UK. Abstract P156.

[6] Margolis AM, Heverling H, Pham PA, Stolbach A (2014). A review of the toxicity of HIV medications. J Med Toxicol.

[7] Langebeek N, Gisolf EH, Reiss P, Vervoort SC, Hafsteinsdottir TB, Richter C, Sprangers MA, Nieuwkerk PT (2014). Predictors and correlates of adherence to combination antiretroviral therapy (ART) for chronic HIV infection: a meta-analysis. BMC Med.

[8] Nakagawa F, Miners A, Smith CJ, Simmons R, Lodwick RK, Cambiano V, Lundgren JD, Delpech V, Phillips AN (2015). Projected lifetime healthcare costs associated with HIV infection. PLoS One.

[9] Arribas JR, Girard PM, Landman R, Pich J, Mallolas J, Martinez-Rebollar M, Zamora FX, Estrada V, Crespo M, Podzamczer D (2015). Dual treatment with lopinavir-ritonavir plus lamivudine versus triple treatment with lopinavir-ritonavir plus lamivudine or emtricitabine and a second nucleos(t)ide reverse transcriptase inhibitor for maintenance of HIV-1 viral suppression (OLE): a randomised, open-label, non-inferiority trial. Lancet Infect Dis.

[10] Di Giambenedetto S, Fabbiani M, Quiros Roldan E, Latini A, D'Ettorre G, Antinori A, Castagna A, Orofino G, Francisci D, Chinello P (2017). Treatment simplification to atazanavir/ritonavir + lamivudine versus maintenance of atazanavir/ritonavir + two NRTIs in virologically suppressed HIV-1-infected patients: 48 week results from a randomized trial (ATLAS-M). J Antimicrob Chemother.

[11] Fabbiani M, Di Giambenedetto S, Poli A, Borghetti A, Castagna A, Mondi A, Galizzi N, Maillard M, Gori A, Cauda R (2016). Simplification to a dual regimen with darunavir/ritonavir plus lamivudine or emtricitabine in virologically-suppressed HIV-infected patients. J Infect.

[12] Perez-Molina JA, Rubio R, Rivero A, Pasquau J, Suarez-Lozano I, Riera M, Estebanez M, Palacios R, Sanz-Moreno J, Troya J (2017). Simplification to dual therapy (atazanavir/ritonavir + lamivudine) versus standard triple therapy [atazanavir/ritonavir + two nucleos(t)ides] in virologically stable patients on antiretroviral therapy: 96 week results from an open-label, non-inferiority, randomized clinical trial (SALT study). J Antimicrob Chemother.

[13] Pulido F, Ribera E, Lagarde M, Perez-Valero I, Palacios R, Iribarren JA, Payeras A, Domingo P, Sanz J, Cervero M, et al. Dual therapy with darunavir and ritonavir plus lamivudine versus triple therapy with darunavir and ritonavir plus tenofovir disoproxil fumarate and emtricitabine or abacavir and lamivudine for maintenance of HIV-1 viral suppression: randomised, open label, non-inferiority DUAL-GESIDA 8014-RIS-EST45 trial. Clin Infect Dis. 2017;65(12):2112–18. 10.1093/cid/cix734 29020293

[14] Marzolini C, Gibbons S, Khoo S, Back D (2016). Cobicistat versus ritonavir boosting and differences in the drug-drug interaction profiles with co-medications. J Antimicrob Chemother.

[15] Joly V, Burdet C, Landman R, et al. Promising results of dolutegravir + lamivudine maintenance in ANRS 167 LAMIDOL Trial. 24th Conference on Retroviruses and Opportunistic Infections. 13–16 February 2017, Seattle, WA, USA. Abstract 458.

[16] Kelly SG, Nyaku AN, Taiwo BO (2016). Two-drug treatment approaches in HIV: finally getting somewhere?. Drugs.

[17] Maggiolo F, Gulminetti R, Pagnucco L, Digaetano M, Benatti S, Valenti D, Callegaro A, Ripamonti D, Mussini C (2017). Lamivudine/dolutegravir dual therapy in HIV-infected, virologically suppressed patients. BMC Infect Dis.

[18] Reynes J, Meftah N, Tuaillon E, et al. Dual regimen with Dolutegravir and Lamivudine maintains virologic suppression even in heavily treatment experienced HIV-infected patients: 96 weeks results from maintenance DOLULAM study. 9th IAS Conference on HIV Science. 23–26 July 2017, Paris, France. Abstract MOPEB0322. .

[19] Cahn P, Rolòn MJ, Figueroa MI, et al. Dolutegravir-lamivudine as initial therapy in HIV-infected, ARV naive patients: 48 week results of the PADDLE trial. 21st International AIDS Conference. 18–22 July 2016, Durban, South Africa. Abstract FRAB0104LB. 10.7448/IAS.20.01.21678 PMC5515053 28537061

[20] Oliveira M, Ibanescu RI, Pham HT, Brenner B, Mesplede T, Wainberg MA (2016). The M184I/V and K65R nucleoside resistance mutations in HIV-1 prevent the emergence of resistance mutations against dolutegravir. AIDS.

[21] J.A. Perez-Molina, F. Pulido, S. Di Gianbenedetto, et al. Individual patient data meta-analysis of randomized controlled trials of dual therapy with a boosted protease inhibitor plus lamivudine for maintenance of virological suppression Gesida study 9717. EACS - 16th European AIDS Conference, 25–27 October 2017, Milan, Italy. Abstract PS1/1.

[22] Llibre JM, Hung C, Brinson C (2018). Efficacy, safety, and tolerability of dolutegravir-rilpivirine for the maintenance of virological suppression in adults with HIV-1: phase 3, randomised, non-inferiority SWORD-1 and SWORD-2 studies. Lancet.

[23] Taiwo BO, Marconi VC, Berzins B, et al. Dolutegravir plus lamivudine maintain HIV-1 suppression through week 48 in a pilot randomized trial. Clin Infect Dis. 2017;26 [Epub ahead of print]. 10.1093/cid/cix1131 PMC5961309 29293895

[24] Rossetti B, Montagnani F, De Luca A. Current and emerging two-drug approaches for HIV-1 therapy in ART-naïve and ART-experienced, virologically suppressed patients. Expert Opin Pharmacother 2018 Apr 20:1–26. 10.1080/14656566.2018.1457648 29676935

[25] Soriano V, Fernandez-Montero JV, Benitez-Gutierrez L (2017). Dual antiretroviral therapy for HIV infection. Expert Opin Drug Saf.

[26] Bagnis CI, Stellbrink HJ. Protease inhibitors and renal function in patients with HIV infection: a systematic review. Infect Dis Ther. 2015;4(1):15–50. 10.1007/s40121-014-0056-4 PMC4363218 25567681

[27] Shah BM, Schafer JJ, Desimone JA, Jr. Dolutegravir: a new integrase strand transfer inhibitor for the treatment of HIV. Pharmacotherapy 2014, 34(5):506–520. 10.1002/phar.1386 24347095

